# Supplementary material for: Microvascular flow alterations in critically ill COVID-19 patients: A prospective study
Source: PLoS One. 2021 Feb 8;16(2):e0246636. doi: 10.1371/journal.pone.0246636 (PMC7870020; doi:10.1371/journal.pone.0246636)
Supplement: Attachment — Submitted filename: reveiwer answer.docx [file pone.0246636.s001.docx]

Dear editor,

Please find a revised version of our manuscript entitled “**Microvascular flow alterations in critically ill COVID-19 patients: a prospective study**”.

We would like to thank the editor for the opportunity to revise the manuscript. We have thoroughly revised the manuscript on the basis of the reviewer comment.

In summary, we believe that we have addressed all the comments and have markedly improved the manuscript.

Yours sincerely,

Osama Abou-Arab, MD PhD and Yazine Mahjoub, MD PhD

Additional Editor Comments:

Please refer to the reviewers' comments and revise your manuscript as appropriate. Keep safe and all the best.

Journal Requirements:

When submitting your revision, we need you to address these additional requirements.

**1. Please ensure that your manuscript meets PLOS ONE's style requirements, including those for file naming. The PLOS ONE style templates can be found at**

https://journals.plos.org/plosone/s/file?id=wjVg/PLOSOne_formatting_sample_main_body.pdf and

https://journals.plos.org/plosone/s/file?id=ba62/PLOSOne_formatting_sample_title_authors_affiliations.pdf

**2.Thank you for including your ethics statement:**

"According to French law on clinical research [11], the study was declared and registered at the Commission Numérique Informatiques et Libertés (registration identifier:

I2020_843_0026) and was registered on ClinicalTrial.gov on 21st of April 2020 (identifier: NCT04354558). This study was conducted in accordance with the amended Declaration of Helsinski."

We thank the editor for his comment and made changes as suggested

a. Please amend your current ethics statement to include the full name of the ethics committee/institutional review board(s) that approved your specific study.

b. Once you have amended this/these statement(s) in the Methods section of the manuscript, please add the same text to the “Ethics Statement” field of the submission form (via “Edit Submission”).

For additional information about PLOS ONE ethical requirements for human subjects research, please refer to http://journals.plos.org/plosone/s/submission-guidelines#loc-human-subjects-research.

**3. In the Methods, please state:**

**- Why written consent could not be obtained**

**- Whether the Institutional Review Board (IRB) approved use of oral consent**

**- How oral consent was documented**

We thank the editor for his comment and made changes as follows:

Our study was classified as category 3 by our local IRB, which implies to inform the patient of his rights to use his data (doi: 10.1016/j.accpm.2020.10.012).

We added details in the method section as follows: Regarding French laws, our study was classified as a category 3 [11]. Thus, written consent was waved. The patient was orally informed in his right to oppose to the use of his data after delivering a written information on the study. A copy of the written information with the patient name was added to the medical record.

Written consent was waived in line with the French laws and with the local IRB decision.

To document the oral consent, a copy of the written information with the patient name and the date was joined to the medical record.

We added a sentence as follows: A copy of the written information with the patient name was joined to the medical record.

For more information, please see our guidelines for human subjects research: https://journals.plos.org/plosone/s/submission-guidelines#loc-human-subjects-research

**4. Please provide a sample size and power calculation in the Methods, or discuss the reasons for not performing one before study initiation.**

Concerning the sample size, given the absence of preexisting data on MFI or others Cytocam parameters in Covid-19, we included the maximum of patients we could.

We made changes in the method section.

**5. Thank you for stating the following financial disclosure:**

**"NO- The funders had no role in study design, data collection and analysis, decision to publish, or preparation of the manuscript."**

The study was not funded.

**At this time, please address the following queries:**

**Please clarify the sources of funding (financial or material support) for your study. List the grants or organizations that supported your study, including funding received from your institution.**

The study was not funded.

**State what role the funders took in the study. If the funders had no role in your study, please state: “The funders had no role in study design, data collection and analysis, decision to publish, or preparation of the manuscript.”**

**If any authors received a salary from any of your funders, please state which authors and which funders.**

**If you did not receive any funding for this study, please state: “The authors received no specific funding for this work.”**

We added the mentioned state in the manuscript.

**Please include your amended statements within your cover letter; we will change the online submission form on your behalf.**

**6. We note that you have indicated that data from this study are available upon request. PLOS only allows data to be available upon request if there are legal or ethical restrictions on sharing data publicly. For information on unacceptable data access restrictions, please see http://journals.plos.org/plosone/s/data-availability#loc-unacceptable-data-access-restrictions.**

**In your revised cover letter, please address the following prompts:**

**a) If there are ethical or legal restrictions on sharing a de-identified data set, please explain them in detail (e.g., data contain potentially identifying or sensitive patient information) and who has imposed them (e.g., an ethics committee). Please also provide contact information for a data access committee, ethics committee, or other institutional body to which data requests may be sent.**

There is French legal restrictions.

We added the following statement on data sharing

“All relevant data are within the paper. Raw data are available after notification and authorization of the competent authorities. In France, all computer data (including databases, in Cover Letter particular patient data) are protected by the National Commission on Informatics and Liberty (CNIL), the national data protection authority for France. CNIL is an independent French administrative regulatory body whose mission is to ensure that data privacy law is applied to the collection, storage, and use of personal data. As the database of this study was authorized by the CNIL, we cannot make available data without prior agreement of the CNIL. Requests may be sent to: [picard.carl@chu-amiens.fr](mailto:picard.carl@chu-amiens.fr)”

**b) If there are no restrictions, please upload the minimal anonymized data set necessary to replicate your study findings as either Supporting Information files or to a stable, public repository and provide us with the relevant URLs, DOIs, or accession numbers. Please see http://www.bmj.com/content/340/bmj.c181.long for guidelines on how to de-identify and prepare clinical data for publication. For a list of acceptable repositories, please see http://journals.plos.org/plosone/s/data-availability#loc-recommended-repositories.**

**We will update your Data Availability statement on your behalf to reflect the information you provide.**

**7. Please ensure that you refer to Figure 1 and 2 in your text as, if accepted, production will need this reference to link the reader to the figure.**

We cited Figure 1 and 2 in the results section when appropriate.

[Note: HTML markup is below. Please do not edit.]

Reviewers' comments:

Reviewer's Responses to Questions

Comments to the Author

1. Is the manuscript technically sound, and do the data support the conclusions?

The manuscript must describe a technically sound piece of scientific research with data that supports the conclusions. Experiments must have been conducted rigorously, with appropriate controls, replication, and sample sizes. The conclusions must be drawn appropriately based on the data presented.

Reviewer #1: Yes

Reviewer #2: Yes

2. Has the statistical analysis been performed appropriately and rigorously?

Reviewer #1: Yes

Reviewer #2: Yes

3. Have the authors made all data underlying the findings in their manuscript fully available?

The PLOS Data policy requires authors to make all data underlying the findings described in their manuscript fully available without restriction, with rare exception (please refer to the Data Availability Statement in the manuscript PDF file). The data should be provided as part of the manuscript or its supporting information, or deposited to a public repository. For example, in addition to summary statistics, the data points behind means, medians and variance measures should be available. If there are restrictions on publicly sharing data—e.g. participant privacy or use of data from a third party—those must be specified.

Reviewer #1: Yes

Reviewer #2: Yes

4. Is the manuscript presented in an intelligible fashion and written in standard English?

PLOS ONE does not copyedit accepted manuscripts, so the language in submitted articles must be clear, correct, and unambiguous. Any typographical or grammatical errors should be corrected at revision, so please note any specific errors here.

Reviewer #1: Yes

Reviewer #2: Yes

5. Review Comments to the Author

Please use the space provided to explain your answers to the questions above. You may also include additional comments for the author, including concerns about dual publication, research ethics, or publication ethics. (Please upload your review as an attachment if it exceeds 20,000 characters)

Reviewer #1: The authors showed interesting microcirculatory patterns in COVID-19 patients. I have some comments and questions:

**1) The authors mentioned that sublingual microcirculation was assessed within 48 hours after ICU admission. What about the median inclusion time?**

**Answer:** We thank the reviewer for his comment. We checked for ICU exact admission time on our electronic health record software and match it with the Cytocam software record for each patient.

The median time inclusion was similar between group, around 20 hours. We added it in table 2 as follows:

| Time from ICU admission to inclusion; *hours* | 20 [5-32] | 21 [6-30] | 0.682 |
| --- | --- | --- | --- |

**Comment: Did sublingual microcirculatory assessment was faster in critical group than severe group? (p.7)**

**Answer:** Regarding the assessment, we proceeded with the same manner whatever the patient severity. We took the necessary time to record 5 videos of good quality as required by the semi-automatic software analysis. In cases of bad quality of videos, we tried as many attempts as necessary to obtain 5 videos of good quality. We did not specifically record the assessment duration. However, it seems to the operator that the duration of the assessment was faster in sedated patients compared to awake patients.

2) **Comment For data collection for systemic hemodynamic variables, Was cardiac output measured by echocardiography in all patients ? if yes , was echocardiography performed by 1 persons due to operator dependent? For capillary refill time, could you mention how did you performed capillary refill time by eye ball or using chronometer ?**

**Answer:** We thank the reviewer for his comment.

Cardiac output was measured by Doppler echocardiography. All measurements for all patients were performed by the same operator (CB). We added a sentence in the method section: “Cardiac output was measured by Doppler echocardiography by the same operator(CB).”

Regarding capillary refill time, measurements were standardized using a chronometer. We added details on the standardization of measurement as follows:

“Capillary refill time was performed as follows: a firm pressure was applied to the surface of the index finger distal phalanx. The pressure was increased until the skin became blank and maintained for 10s. The pressure was then released. The time for return to pre-existent skin color was measured using a chronometer [14].”

**3) For demographic data, were SOFA and SAPS II scores calculated at ICU admission or time of inclusion ? It would be better if you mention severity scores at inclusion time.**

**Answer:** We agree with reviewer. SAPS II and SOFA were calculated at inclusion time (time of microcirculation assessment) as mentioned in data collection section. We added “at inclusion” in the results section and in Table 1.

**Comment: Was PaO2/FiO2 ratio in severe group different from patients in critical group.**

**Answer:** We thank the reviewer for his relevant comment. However, in the severe group patients had a facial mask or a nasal canula which makes the exact estimation of FiO2 difficult. That is why we apologize for not being able to report this value for the severe group.

**Comment: The authors mentions number of patients who required vasopressor, in these patients, were they shock? Could you possibly mention numbers of patients with shock and without shock and did microcirculatory profiles differ between patients with and without shock.**

**Answer:** We thank the reviewer for his relevant comment. No patient had a shock state at inclusion. As reported in table 2, the dose of norepinephrine was quite low (0.02 μg.kg.min^-1^). Macro hemodynamics parameters were similar regarding cardiac output and heart rate with no septic shock pattern. Vasopressors use was probably related to sedation drugs (propofol) used to ensure mechanical ventilation.

**4) Was the microcirculatory profiles related to lung recovery in critical group?**

**Answer:** We thank the reviewer for his relevant comment. Unfortunately, only one time point measurement was performed. We agree that several a time point monitoring of microcirculation during the stay would had been very interesting. However, microcirculatory record was performed shortly after initiation of mechanical ventilation( i.e: worsening of lung function).

**5) Did microcirculatory alter in patients with COVID19 compare with healthy volunteers?**

**Answer:** We thank the reviewer for his relevant comment.

The guidelines on sublingual assessment (doi: 10.1007/s00134-018-5070-7) described the convective property according to the red blood cell velocities (explored by the MFI and the PVD), the diffuse capacity (explored by the TVD) and the heterogeneity in perfusion (HI)

No reference values were admitted but healthy subjects values are available from previous studies (doi: 10.1097/CCM.0b013e31823dae59).

In a healthy subject, a normal MFI is expected to be over 2.6 which was the case for our critical group but not in the severe group.

PVD in healthy subject was reported to be about 15 mm/mm2. PVD was less decreased in the severe group.

To summarize, the critical group tend to have higher red blood velocities when comparing the severe group (PVD and MFI higher)

In a healthy subject, TVD is around 15 mm/mm2 and HI around 0.03. In our population, these parameters were close to healthy subjects’ values. Hence, in our cohort COVID-19 patients did not seem to have convective impairment.

**Reviewer #2: I would like to congratulate the authors for their courage and enthusiasm in performing such time consuming and labor intensive study protocol in the ICU.**

**Answer: we are very grateful to the reviewer for his consideration and encouragements.**

I have just minor concerns:

**1. The title could be more focussed beginning by deleting the word "systemic". Please rewrite the title specifically by adding more microcirculatory items like capillary, microvascular flow, alterations etc...**

**Answer:** We thank the reviewer for his helpful comment. We changed the title as follows: “Microvascular flow alterations in critically ill COVID-19 patients: a prospective study.”

**2. Please write COVID-19 with capital letters everywhere.**

**Answer:** We made changes in the manuscript.

**3. Abstract: Conclusion should be specified by replacing "better microcirculatory pattern" to more focussed description. What is better???? What is still wrong in the microcirculation???**

**Answer:** We thank the reviewer for his comment and made changes to better describe the profiles. We made changes as follows:

Critical COVID-19 patients under mechanical ventilation seem to have higher red blood cell velocity than severe non-ventilated patients.

**4. Introduction: "Microcirculation pattern of Covid-19 might differ from other sepsis causes" could implicate that COVID-19 is (pre)sepsis. Please rewrite this sentence that doesn't confuse sepsis with this new disease.**

“Timing for intubation and invasive ventilation in COVID-19 patients is a subject of debate [6]. In this new disease, microcirculatory impairment might explain disease progression and differences between invasively and non-invasively ventilated patients. [3]

**5. Methods: MFI=microvascular flow index**

**Answer:** We apologize for this mistake and made changes

**6. Results: Please refer to figure 1 and 2 for the responsible data.**

**Answer:** We made changes and referred to figure 1 and 2 when required in the result section.

**7. Discussion: First sentence is very superficial. Higher microcirculation parameters does mean nothing. Please redifine this e.g. hyperdynamic at least?? High density vasculary flows? high red blood cell velocity?**

**Answer:** We thank the reviewer for his relevant comments and hope we better describe the profiles.

Here we made the following changes.

“Our findings suggest that the critical group had higher red blood cell velocity (higher MFI values) and a better vessel density (higher DBS and PVD). Capillary diffuse properties seem to be preserved given the values of PPV, TVD and HI.”

**8. From limitations you could delete the third limitation hence there are thousands of studies which support the idea of sublingual microcirculation as the best paramter for follow-up of the end-organ perfusion ("systemic (micro)circulation").**

**Answer:** We made changes and removed the sentence.

**9. Finaly add in your discussion the more advanced novel automated microcirculation analysis which could be used for more detailed and accurate microcirculatory analysis published by Hilty et al. Crit Care Med 2020. Novel studies with novel and efficient microcirculation analysis programs could make the clinicians ICU life scientifically comfortable.**

**Answer:** We thank the reviewer for his comment and added a sentence explaining the MicroTools.

“A new tool called MicroTools software allowing an automated video analysis have been recently validated in a large international retrospective database [29]. This kind of automated analysis allowing resuscitation therapies targeted on microcirculatory parameters should be applied in further investigations.”

**Comments**

**Tables: Please add more clinical data into the tables**

**- CT lungs for detecting pulmonary embolism; how many performed how many positive in each group?**

**- Day of illness?**

**- Day of intubation?**

**- Day of optiflow during the microcirculation measurement?**

**Answer:** We thank the reviewer for his comment and completed Table 1 and Table 2 as follows.

**- What was the anticoagulation strategy? Profylaxes, therapeutic? which agent? UFH? LMWH? What was the follow-up/monitoring strategy for coagulation?**

**Answer: during the first wave of COVID-19 pandemic,** our local institutional protocol included for all COVID-19 patients admitted to ICU, a therapeutic anticoagulation strategy with unfractioned heparin to achieve anti Xa activity between 0.5 and 0.8 UI/ml.

UFH was started at ICU admission. Anti Xa activity was checked daily and 4 hours after each dose change. We added a sentence in the method section “All COVID-19 patients were under unfractionned heparin to achieve anti Xa activity between 0.5 and 0.8 UI/ml”
